# Association between the use of epidural analgesia during labour and incidence of postpartum depression

**DOI:** 10.1371/journal.pone.0289595

**Published:** 2023-10-31

**Authors:** Hanin Mohammed Yaqoob Ahmad, Lama Adnan Althagafi, Ghazal Zuhair Albluwe, Shahd Mohammed Kadi, Relam Ibrahim Alhassani, Nedaa Mohammed Bahkali

**Affiliations:** 1 Medical Students at King Abdulaziz University, Jeddah, Saudi Arabia; 2 Medical Intern at King Abdulaziz University, Jeddah, Saudi Arabia; 3 Obstetrics and Gynecology, Assistant professor in King Abdulaziz University, Jeddah, Saudi Arabia; Fondazione Policlinico Universitario Agostino Gemelli IRCCS, Universita’ Cattolica del Sacro Cuore, ITALY

## Abstract

**Introduction:**

Postpartum depression is a significant episode of depression beginning after giving birth. The prevalence of postpartum depression is approximately 20% in Jeddah, Saudi Arabia. Epidural analgesia is the gold standard for labour pain management. Conflicting results exist regarding the association between postpartum depression and epidural analgesia use during labour. Accordingly, this study assessed the association between epidural analgesia use and postpartum depression incidence.

**Methods:**

A prospective observational study of 170 mothers was conducted, with surveys administered after labour and at six weeks postpartum. Surveys included the following: mothers’ demographics, obstetric history, postpartum depression (Edinburgh Postnatal Depression Scale), and pain severity (Visual Analogue Scale).

**Results:**

In the final analysis, 91 patients were enrolled. Epidural analgesia was administered to 48.4% of mothers during labour. Nearly two-thirds of mothers learned about EA via sources including family members and social media. However, more than half reported worries regarding epidural analgesia. Edinburgh Postnatal Depression Scale scores showed that 38 mothers (41.8%) likely had depressive symptoms within two days following delivery. Further, 35 (38.5%) met criteria for postpartum depression at six weeks postpartum. For both groups regardless use of analgesia, the mean Visual Analogue Scale score at two days postpartum was 4.16 ± 2.13. Data revealed no correlation between epidural analgesia use and Edinburgh Postnatal Depression Scale within two days and at six weeks postpartum. Multiple regression analysis showed Edinburgh Postnatal Depression Scale scores correlated with Visual Analogue Scale scores but not epidural analgesia use at 1–2 days postpartum.

**Conclusion:**

This study showed that depressive symptoms resolved in three percent of participants. This suggests that institutions should increase postpartum depression awareness during the antenatal period and implement effective post-delivery screening systems for postpartum depression.

## Introduction

Postpartum depression [PPD] is an episode of major depression starting within four weeks of giving birth [1]. Symptoms of PPD include but are not limited to depressed mood, inability to feel pleasure, overwhelming guilt, insomnia, changes in appetite, diminished concentration, irritability, anxiety, psychomotor agitation or retardation, and suicidal ideation [1, 2]. Numerous risk factors are associated with PPD such as social stress, marital status, family support, and genetic predisposition [3, 4]. Depressive disorders have many devastating effects including a mother’s refusal to breastfeed and poor mother-infant bonding [5]. PPD is common, affecting mothers and families [6]. It has been shown that 17.22% of all pregnant women have PPD globally [7]. Among them, Middle Eastern and Asian mothers are more likely to suffer from PPD [8]. In Jeddah, Saudi Arabia, the prevalence of PPD among women is approximately 20% [9].

Epidural Analgesia [EA] is the gold standard and most efficient technique for labour pain management [10–12]. Hence, its use is expanding globally [13]. EA is a central nerve blockade technique that involves the injection of a local anaesthetic into the lower region of the back close to the nerves that transmit pain of the contracting the uterus and birth canal [14]. EA improves maternal satisfaction and decreases maternal pain and stress [10, 15, 16]. Many studies have estimated an association between EA and PPD. A study performed in the United States showed that EA-mediated pain improvement was associated with a reduced risk of PPD [17]. In addition, a Chinese prospective observational study revealed that EA use reduced PPD risk [18]. Furthermore, a study performed in Singapore concluded that compared to women who chose non-EA during birth, those who received EA had a considerably decreased risk of PPD [19]. Similarly, a recent study in Istanbul, Turkey that enrolled almost 100 pregnant women showed that mothers who received EA had a smaller pain scale score, and therefore, lower risk of PPD compared to mothers not received EA [20]. In contrast, several prospective cohort studies conducted in Singapore and the United States failed to demonstrate an association between PPD and labour EA [21, 22]. Eckerdal et al. [2020] at Uppsala University Hospital, Sweden, confirmed that after adjusting for potential confounders [age, fear of childbirth, and prenatal depression symptoms], EA use did not increase risk of PPD at 6 weeks postpartum [23].

Despite prior research done on the topic, it remains unclear whether EA use affects PPD incidence. Accordingly, this study aimed to assess the association between EA use and PPD among pregnant women treated at King Abdulaziz University Hospital from June to November 2022.

## Materials and methods

### Study design and setting

This prospective observational study was conducted at King Abdulaziz University Hospital [KAUH], Jeddah, Saudi Arabia, in the Obstetrics and Gynecology Department from June to November 2022. Women who had spontaneous vaginal delivery [SVD] with or without EA, who consented verbally and documented their consent in electronic form, were included. Participants who were previously diagnosed with psychiatric illness and non-Arabic speakers were excluded.

### Sample size and sampling procedure

**Fig 1:**
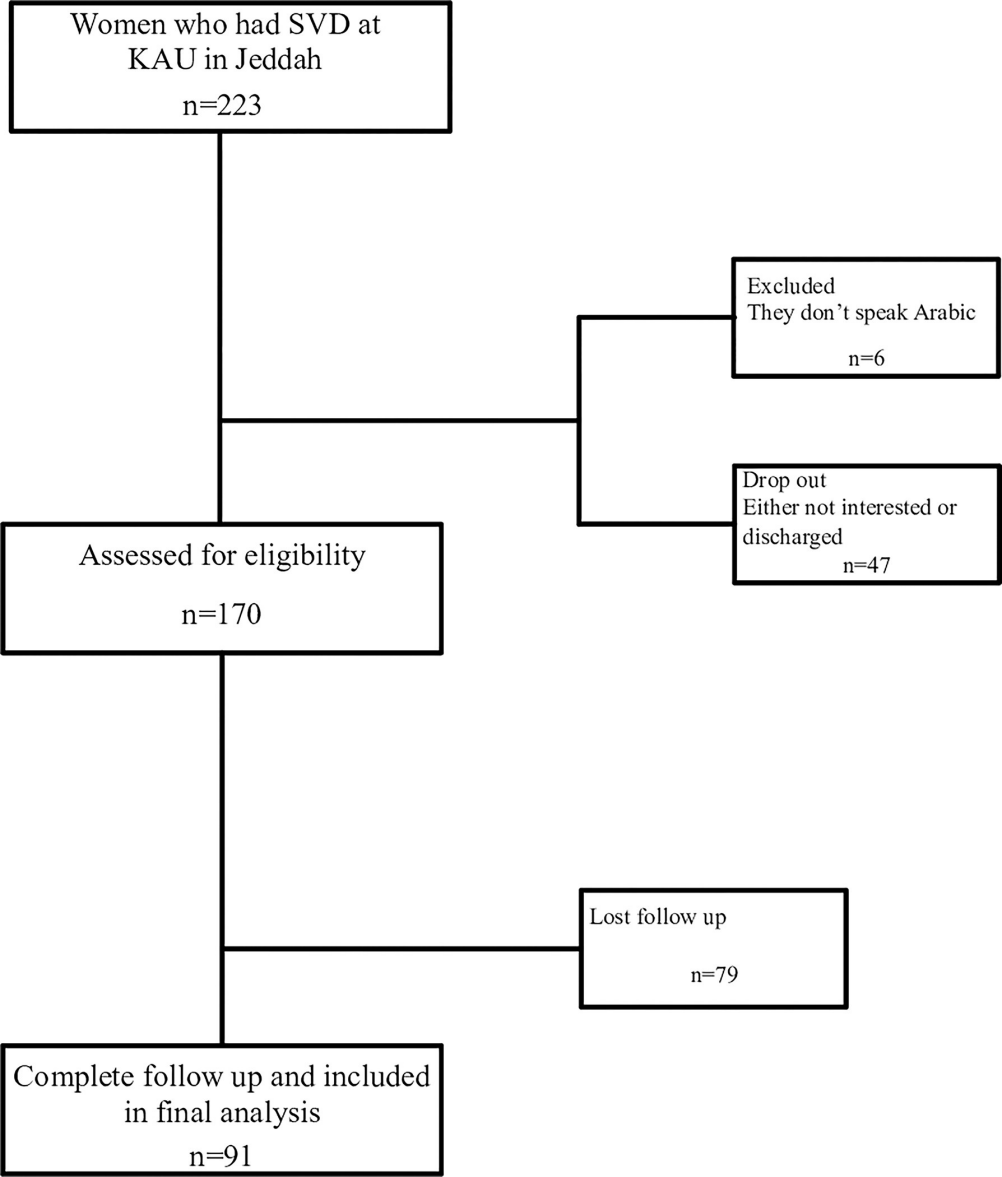


A total of 170 mothers were initially interviewed by the research team during their first days postpartum. Among them, 91 participants were enrolled in the final analysis completing a survey at 6-weeks postpartum. Mothers included in the study were aged between 19–41 years, delivered via SVD, and may or may not have used EA. Forty-four mothers had EA during delivery, while forty-seven did not (Fig 1).

### Data collection

All participants were informed regarding study objectives and confidentiality. Verbal consent was obtained from all the participants at the beginning of interviews. Data were collected using an Arabic survey using Google Forms with the guidance of researchers. To be included in the final analysis, the survey would have to be answered twice, between 24 and 48 hours post delivery and at 6 weeks postpartum at the end of the follow-up period. All participants were informed by the research team that they would need to complete a survey after 6 weeks; therefore, phone numbers were requested to reassure participation to fill the survey at the end of the follow-up period.

The survey consisted of four main sections:1] patient demographics, 2] full obstetric history, 3] Edinburgh Postnatal Depression Scale [EPDS], and 4] Visual Analogue Scale [VAS] at 1–2 days post delivery. Demographic data collected included age, educational level, occupation, and household income. Obstetric history included the following items: gravidity, parity, number of abortions, worries regarding the experience of labour and motherhood, use of EA, any concerns about EA, did the patient read more about EA, and was EA use self-requested or recommended by a physician. A valid Arabic version of the Edinburgh Postnatal Depression Scale [EPDS] was used, which consists of 10 screening questions, each scored 4 points [0–3], with a minimum score of 0 and a maximum score of 30 [24]. The cutoff score for depressive symptoms and PPD was ≥ 10. Any thoughts of suicide were further evaluated despite the patient’s total score. The final section of the assessment was a pain assessment using the Visual Analogue Scale [VAS], a subjective pain rating scale in which the lowest intensity pain is indicated by 0 and the highest by 10.

### Statistical analysis

Microsoft Excel 2016 [Microsoft Corporation, Microsoft 365. Riyadh, SA] was used for data entry and analysis using IBM SPSS Statistics version 26. Independent sample T-test and correlation test were used to measure the association between two variables with 95% CI. In addition, One-way ANNOVA test were used to measure the association between three variables with 95% CI. All tests had a cutoff of p-value equal or less than 0.05 for to be significant.

### Research ethics

The study was approved by the Research Ethics Committee of KAUH [Ref No:10–22. Written Consent were documented via survey from all participants after explaining the objectives and aims of the study.

## Results

### Demographic analysis

This study aimed to assess the association between EA and PPD. A total of 170 women were initially deemed eligible to participate in the study. After excluding mothers who were lost to follow-up, 91 participants were included in the final analysis, with a mean age of 30.38 ± 5.09 years. The majority of enrolled participants were aged 25–35 years (66%). Patients were divided into the following age groups: < 25 years, 25–35 years, > 35 years, containing 19%, 66%, and 15% of patients, respectively. EA was administered to 44 mothers [48.4%] during labour, while 47 mothers (51.6%) did not undergo EA. Regarding obstetric history, gravidity, parity, and abortion occurred in 3, 3, and 1 cases, respectively. Among participants, 60.4% had a bachelor’s degree and 82.4% were unemployed [Table 1 for demographic data]. Nearly two-thirds were provided information regarding EA via various sources including family members, friends, and social media. Although 52.7% reported concerns regarding epidural use, 37.4% requested EA. In addition, 80.2% experienced fear of delivery [Table 2 for sociodemographic data].

**Table 1:** Participant demographics [n = 91].

Demographic characteristic	
Age, years	30.4 ± 5.1
Gravidity, n	3.4 ± 1.7
Parity, n	2.7 ± 1.4
Abortion, n	1 ± 1
VAS score	4.16 ± 2.12
EPDS score [24–48 hours PP]	9 ± 5.46
EPDS score [6 weeks PP]	8.52 ± 6.26
EA use, n	44, 48.8%

VAS, Visual Analogue Scale; EPDS, Edinburgh Postnatal Depression Scale; EA, epidural analgesia, PP, Postpartum

**Table 2:** Sociodemographic characteristics of patients.

Sociodemographic characteristic		Frequency	Percentage [%]
Age [years]	18–25	17	18.7%
	25–35	60	65.9%
	> 35	14	15.4%
Educational level	Elementary	4	4.4%
	Middle	5	5.5%
	High	24	26.4%
	Bachelor ‘s	55	60.4%
	PhD	1	1.1%
	Master’s	2	2.2%
Employment	Yes	16	17.6%
	No	75	82.4%
Requested an epidural	Yes	34	37.4%
	No	48	52.7%
	Doctor suggestion	9	9.9%
Got advice or read about epidural analgesia [family, friends, or social media]	Yes	61	67%
	No	30	33%
Had worries regarding epidural analgesia	Yes	48	52.7%
	No	43	47.3%
Delivery fears	Yes	73	80.2%
	No	18	19.8%
Caring worries	Yes	40	44%
	No	51	56%

VAS, Visual Analogue Scale; EPDS, Edinburgh Postnatal Depression Scale; EA, epidural analgesia, PP, Postpartum

### Univariate analysis

EPDS scores indicated that 38 mothers [41.8%] likely had depressive symptoms 1–2 days post-delivery, 17 of whom underwent EA. Subsequently, 35 mothers [38.5%] met criteria for PPD 6-weeks postpartum, 15 of whom underwent EA. The survey question, “the thoughts of harming myself has occurred to me”, was answered never in 88 cases, hardly ever in one case, sometimes in one case, and yes quite often in one case. Regarding pain, the mean VAS score of the study population during first two days post-delivery was 4.16 ± 2.13. Moreover, mothers who did and did not undergo EA had mean of pain scores of 4.27 ± 2.45 and 4.06 ± 1.78, respectively. Only two participants reported a VAS score of 10.

### Bivariate analysis

Relationships between demographic data and EA, EPDS and VAS were assessed [Table 3]. Data revealed using intendent sample T-test that no significant relationship between EA use and EPDS score both at 1–2 days and six weeks postpartum [p-values of 0.564 and 0.2, respectively]. Furthermore, correlation test illustrated no statistically significant association between EPDS score and age, gravidity, parity, or abortion was reported. Regarding pain, there was no correlation between VAS scores and epidural use [p = 0.646]. EPDS and VAS scores were significantly correlated [p = 0.0 at two days postpartum]. Moreover, VAS score and age were not significantly associated [p-value of 0.208] calculated via correlation test. In addition, correlation test of gravidity and parity were assessed with VAS [p-values of 0.934 and 0.663, respectively].

**Table 3:** ERDS, EPDS, and VAS scores according to patient characteristics.

Patient characteristic	EPDS [24–48 hours PP], p-value	EPDS [6 weeks PP], p-value	VAS [4.16 ± 2.12], p-value
Age	0.44	0.66	0.21
Gravidity	0.59	0.89	0.93
Parity	0.19	0.47	0.66
Abortion	0.29	0.33	0.57
EA use	0.56	0.32	0.65
	EPDS [24–48 hours PP], p-value	EPDS [6 weeks PP], p-value	VAS [4.16 ± 2.12], p-value
Age	0.44	0.66	0.21
Gravidity	0.59	0.89	0.93
Parity	0.19	0.47	0.66
Abortion	0.29	0.33	0.57
EA use	0.56	0.32	0.65

VAS, Visual Analogue Scale; EPDS, Edinburgh Postnatal Depression Scale; EA, epidural analgesia, PP, Postpartum

Interestingly, an independent sample T-test evaluated a positive relationship between delivery fears [p = 0.006] and caring worries [p = 0.008] with VAS score. The age of the mothers did not influence the decision to request use of EA [p = 0.085]. Age and knowledge about epidurals were not significantly correlated with each other assessed by independent sample T-test [p = 0.265]. In addition, no significant relationship between educational level and EPDS score was observed by independent sample T-test [p = 0.655]. The data illustrated by independent sample T-test that EPDS at two days postpartum was positively associated with caring concerns [p = 0.009] but not with delivery fears [p = 0.095]. Furthermore, an independent sample T-test of EPDS scores at six-weeks postpartum were positively correlated with both delivery fears and caring concerns [p = 0.030 and 0.000, respectively]. Age and birth fears or epidural worries were not significantly correlated from independent sample T-test [p = 0.308 and p = 0.277, respectively]. Bivariate analysis with independent sample T-test revealed that there was no correlation between employment and EPDS score either at 1–2 days or 6 weeks postpartum [p = 0.353 and 0.301, respectively]. Finally, VAS scores did not significantly correlate with age or EA [p = 0.208 and 0.646, respectively].

### Multivariate analysis

Multiple regression analysis used one-way ANOVA revealed that EPDS with VAS scores at 1–2 days postpartum and EA were correlated [p = 0.002].

## Discussion

The current study aimed to assess the association between EA use and the incidence of PPD.

In total, 91 women were enrolled in the final analysis. Similar to studies conducted in Istanbul, the mean age of patients in this study was 30.38 ± 5.09 years [20, 25]. Two thirds of mothers were between 25 and 35 years. This was similar to a previously published Canadian study showed that more than two-thirds of participants were aged 18–35 years [26].

Importantly, 41.8% and 38.5% of EPDS scores reported in the current study were ≥ 10 at 1–2 days and 6 weeks postpartum, respectively. Kaur et al. concluded that percentages of patients with depressive symptoms at 3-days postpartum differed by 2% between mothers received combined spinal and epidural and mothers not received. At 6-weeks postpartum mothers who used analgesia more frequently reported depressive symptoms versus those who did not [27.7% and 16.9%, respectively] [27]. Moreover, the present study showed that at 6-weeks postpartum, EPDS scores demonstrated that 38.5% of mothers likely had PPD. Similarly, in a Chinese study, 48.6% of participants experienced PPD with a higher incidence among mothers not receiving EA [34.6%] [25]. Studies conducted in Canada and the USA reported incidences of PPD of 12.9% and 20%, respectively [22, 26]. Some variability in PPD incidence was observed, even among studies evaluating PPD using the same scale. Notably, PPD occurred despite the use of analgesia, indicating PPD is at least partially associated with other factors.

The present study used VAS [scored from 0 to 10] to estimate pain in women at 1–2 days postpartum. The mean VAS score of mothers undergoing and not undergoing EA was 4.27 ± 2.45 and 4.06 ± 1.78, respectively. In contrast, a recent study conducted in Istanbul that used VAS to estimate pain at 24 hours post-delivery revealed that VAS scores of those given and not given EA were 2.71 ± 0.82 and 5.30 ± 2.26, respectively [20]. In this study, the mean VAS of all study participants was 4.27. This finding is similar to that of another prospective study that reported a mean pain score of 5 for all purturients at the time of cervix dilation to 10-cm [25]. Considering that pain is subjective and assessed at only at a given moment, variance in the measured pain scores is likely.

The current study revealed that EA does not significantly correlate with EPDS score at 1–2 days or 6 weeks postpartum [p = 0.564 and 0.2, respectively]. A similar study conducted in Canada revealed that the use of crude EA is not associated with PPD based on EPDS score at delivery, or 6-weeks or 6-months postpartum [26]. In addition, Tobin et al. and Kaur et al. reported a lack of a correlation between EA and PPD. Tobin et al. screened for PPD at 6–8 days postpartum, while Kaur et al. screened for PPD via EPDS at 3-days and 6-weeks postpartum [22, 27]. On the other hand, a study conducted in China identified a correlation between EPDS score and PPD both at 3-days and 6-weeks post-delivery [25]. Similarly, a study in Singapore concluded that the EPDS score was associated with EA use at 3-months post-delivery [21]. Impressively, a study by Ipek et al. considered that 92 patients revealed no relationship between EA and PPD during the prepartum period; however, a relationship was observed at 6-weeks postpartum [20]. The current study showed that nearly half of the women considered likely had depression, regardless of whether or not they underwent EA, indicating that PPD is a substantial problem.

Here, no association between VAS score and EA use was reported [p = 0.646]. This is in contrast to several studies that reported that EA use resulted in reduced pain severity. One reason that may explain the discrepancy is that different pain scales were used. For example, Ding et al. assessed pain severity using the numerical rating scale [NRS], an 11-point scale used to evaluate pain at various stages of delivery, with 0 indicating no pain at all and 10indicating the worst possible pain [25], while Saadet et al. used the VAS to measure pain intensity [20]. Conflicting results regarding pain associated with epidural likely occur because pain scales subjective measures that may be assessed with different scales and have various influencing factors including gravity incidence, social support, and economic status.

A study conducted in Singapore that assessed the effect of age on PPD prevalence reported that females aged 40–45 years had higher PPD rates than those aged 30–35. This may be due to the fact that older mothers experienced more pregnancy complications than younger mothers [19]. Moreover, Orbach-zinger et al. discovered that older primiparas were at increased risk of PPD versus multiparties of different age groups [28]. Results of the present study are contradictory to those of Orbach-zinger et al.

The present study revealed no correlation between monthly income and educational level versus EPDS score at 1–2 days and 6-weeks postpartum. A prior prospective study illustrated that PPD is correlated with socioeconomic characteristics such as financial stress and maternal income [21]. Socioeconomic status may significantly affect new mothers because they may cause patients to deal with stress and significant life events frequently linked to PPD via different mechanisms.

This research demonstrated that almost 80% of women feared delivery. A cross-sectional study published in 2018 that included 179 women showed that nearly 30% of women feared childbirth based on an assessment using the Fear of Birth Scale [29]. Fearing delivery may be reflective of many factors. For example, mothers giving birth after experiencing the COVID-19 pandemic are likely cautious and tend to worry about economic and health status.

This study found a significant association between the EPDS with VAS scores and EA via a multiple regression analysis. Similarly, authors of the Istanbuli study, Via multiple regression analysis,showed a significant association between EPDS and EA [p = 0.0001] [20]. This is contrary to two studies that reported ny2o significant association between EPDS and pain scores via multivariate analysis [17, 20]. Nevertheless, the literature shows conflicting results regarding the association between EPDS score, and EA use and pain score.

The current study had some limitations. Nearly half of the participants were not included in the final analysis due to loss to follow-up, which resulted in a small sample size. Pain analysis is a subjective measure of pain. Lastly, the study only included patients of King Abdulaziz University Hospital, Jeddah; therefore, results cannot be generalised to the general population. In the future, studies with larger sample sizes should be conducted.

## Conclusion

In conclusion, this study aimed to determine whether there is an association between epidural analgesia and the incidence of PPD among patients treated at KAUH, Jeddah, Saudi Arabia. Data indicate that EA is not associated with the incidence of PPD. Further, EPDS score was not associated with EA at either 1–2 days or 6-weeks postpartum. Furthermore, there was no association between EA use and VAS score. EPDS scores correlated with VAS at 1–2 days postpartum. Importantly 41.8% and 38.5% of patients had EPDS scores indicative of PPD at 1–2 days and 6-weeks postpartum, respectively. Furthermore, only 3% of mothers experienced depressive symptom resolution between 1–2 days and 6-weeks post-delivery. Consequently, institutions should prioritise raising awareness of PPD during the antenatal period and implement an effective post-delivery PPD screening system.

## Supporting information

S1 Checklist STROBE statement—checklist of items that should be included in reports of observational studies. (DOCX)
