# Supplementary material for: Association between the use of epidural analgesia during labour and incidence of postpartum depression
Source: PLoS One. 2023 Oct 31;18(10):e0289595. doi: 10.1371/journal.pone.0289595 (PMC10617733; doi:10.1371/journal.pone.0289595)
Supplement: S1 Checklist — (DOCX) [file pone.0289595.s001.docx]

STROBE Statement—checklist of items that should be included in reports of observational studies

|  | Item No. | Recommendation | Page  No. | Relevant text from manuscript |
| --- | --- | --- | --- | --- |
| **Title and abstract** | 1 | (*a*) Indicate the study’s design with a commonly used term in the title or the abstract | 1 | **Association between the use of epidural analgesia during labour and incidence of postpartum depression: a prospective observational study.** |
|  |  | (*b*) Provide in the abstract an informative and balanced summary of what was done and what was found | 2 | **A prospective observational study of 170 mothers was conducted, with surveys administered after labour and at six weeks postpartum. Surveys included the following: mothers’ demographics, obstetric history, postpartum depression (Edinburgh Postnatal Depression Scale), and pain severity (Visual Analogue Scale). Data revealed no correlation between epidural analgesia use and Edinburgh Postnatal Depression Scale within two days and at six weeks postpartum. The study showed that depressive symptoms resolved in three percent of participants.** |
| Introduction | | | |  |
| Background/rationale | 2 | Explain the scientific background and rationale for the investigation being reported | 4 | **Postpartum depression [PPD] is an episode of major depression starting within four weeks of giving birth. Epidural Analgesia [EA] is the gold standard and most efficient technique for labour pain management. In Jeddah, Saudi Arabia, the prevalence of PPD among women is approximately 20%** |
| Objectives | 3 | State specific objectives, including any prespecified hypotheses | 2 | **To assess the association between Epidural Analgesia and the incidence of Postpartum Depression.** |
| Methods | | | |  |
| Study design | 4 | Present key elements of study design early in the paper | 5 | **A prospective observational study.** |
| Setting | 5 | Describe the setting, locations, and relevant dates, including periods of recruitment, exposure, follow-up, and data collection | 5 | **At King Abdulaziz university hospital, Jeddah, Saudi Arabia in Obstetrics and Gynecology Department from June to November 2022.** |
| Participants | 6 | (*a*) *Cohort study*—Give the eligibility criteria, and the sources and methods of selection of participants. Describe methods of follow-up  *Case-control study*—Give the eligibility criteria, and the sources and methods of case ascertainment and control selection. Give the rationale for the choice of cases and controls  *Cross-sectional study*—Give the eligibility criteria, and the sources and methods of selection of participants | 5 | **This prospective observational study was conducted at King Abdulaziz University Hospital [KAUH], Jeddah, Saudi Arabia, in the Obstetrics and Gynecology Department from June to November 2022. Women who had spontaneous vaginal delivery [SVD] with or without EA, who consented verbally and documented their consent in electronic form, were included. Participants who were previously diagnosed with psychiatric illness and non-Arabic speakers were excluded.**  **A total of 170 mothers were initially interviewed by the research team during their first days postpartum. Among them, 91 participants were enrolled in the final analysis completing a survey at 6-weeks postpartum. Mothers included in the study were aged between 19-41 years, delivered via SVD, and may or may not have used EA. Forty-four mothers had EA during delivery, while forty-seven did not.** |
|  |  | (*b*) *Cohort study*—For matched studies, give matching criteria and number of exposed and unexposed  *Case-control study*—For matched studies, give matching criteria and the number of controls per case | 5 | **A total of 170 mothers were initially interviewed by the research team during their first days postpartum. Among them, 91 participants were enrolled in the final analysis completing a survey at 6-weeks postpartum. Mothers included in the study were aged between 19-41 years, delivered via SVD, and may or may not have used EA. Forty-four mothers had EA during delivery, while forty-seven did not.** |
| Variables | 7 | Clearly define all outcomes, exposures, predictors, potential confounders, and effect modifiers. Give diagnostic criteria, if applicable | 6,7 | **A valid Arabic version of the Edinburgh Postnatal Depression Scale [EPDS] was used, which consists of 10 screening questions, each scored 4 points [0-3], with a minimum score of 0 and a maximum score of 30 [24]. The cutoff score for depressive symptoms and PPD was ≥ 10. Any thoughts of suicide were further evaluated despite the patient’s total score. The final section of the assessment was a pain assessment using the Visual Analogue Scale [VAS], a subjective pain rating scale in which the lowest intensity pain is indicated by 0 and the highest by 10.** |
| Data sources/ measurement | 8* | For each variable of interest, give sources of data and details of methods of assessment (measurement). Describe comparability of assessment methods if there is more than one group | 6,7 | **The survey consisted of four main sections:1] patient demographics, 2] full obstetric history, 3] Edinburgh Postnatal Depression Scale [EPDS], and 4] Visual Analogue Scale [VAS] at 1-2 days post delivery. Demographic data collected included age, educational level, occupation, and household income. Obstetric history included the following items: gravidity, parity, number of abortions, worries regarding the experience of labour and motherhood, use of EA, any concerns about EA, did the patient read more about EA, and was EA use self-requested or recommended by a physician. A valid Arabic version of the Edinburgh Postnatal Depression Scale [EPDS] was used, which consists of 10 screening questions, each scored 4 points [0-3], with a minimum score of 0 and a maximum score of 30. The cutoff score for depressive symptoms and PPD was ≥ 10. Any thoughts of suicide were further evaluated despite the patient’s total score. The final section of the assessment was a pain assessment using the Visual Analogue Scale [VAS], a subjective pain rating scale in which the lowest intensity pain is indicated by 0 and the highest by 10.** |
| Bias | 9 | Describe any efforts to address potential sources of bias |  | **N/A** |
| Study size | 10 | Explain how the study size was arrived at | 6 | **All mothers were completed a survey at 6 weeks postpartum.** |

Continued on next page

| Quantitative variables | 11 | Explain how quantitative variables were handled in the analyses. If applicable, describe which groupings were chosen and why |  | **N/A** |
| --- | --- | --- | --- | --- |
| Statistical methods | 12 | (*a*) Describe all statistical methods, including those used to control for confounding | 7 | **Microsoft Excel 2016[Microsoft Corporation, Microsoft 365. Riyadh, SA] was used for data entry and analysis using IBM SPSS Statistics version 26. Independent sample T-test and correlation test were used to measure the association between two variables with 95% CI. In addition, One-way ANNOVA test were used to measure the association between three variables with 95% CI. All tests had a cutoff of p-value equal or less than 0.05 for to be significant.** |
|  |  | (*b*) Describe any methods used to examine subgroups and interactions |  | **N/A** |
|  |  | (*c*) Explain how missing data were addressed |  | **N/A** |
|  |  | (*d*) *Cohort study*—If applicable, explain how loss to follow-up was addressed  *Case-control study*—If applicable, explain how matching of cases and controls was addressed  *Cross-sectional study*—If applicable, describe analytical methods taking account of sampling strategy | 6 | **A total of 170 mothers were initially interviewed by the research team during their first days postpartum. Among them, 91 participants were enrolled in the final analysis completing a survey at 6-weeks postpartum.** |
|  |  | (*e*) Describe any sensitivity analyses |  | **N/A** |
| Results | | | | |
| Participants | 13* | (a) Report numbers of individuals at each stage of study—eg numbers potentially eligible, examined for eligibility, confirmed eligible, included in the study, completing follow-up, and analysed | 4 | **A total of 170 mothers were initially interviewed by the research team during their first days postpartum.**  **Among them, 91 participants were enrolled in the final analysis.** |
|  |  | (b) Give reasons for non-participation at each stage | 5 | **Participants who were previously diagnosed with psychiatric illness and non-Arabic speakers were excluded.** |
|  |  | (c) Consider use of a flow diagram |  | **N/A** |
| Descriptive data | 14* | (a) Give characteristics of study participants (eg demographic, clinical, social) and information on exposures and potential confounders | 5 | -**Demographic data collected included age, educational level, occupation, and household income.**  **-Obstetric history included the following items: gravidity, parity, number of abortions, worries regarding the experience of labour and motherhood, use of EA, any concerns about EA, did the patient read more about EA, and was EA use self-requested or recommended by a physician.**  **-A valid Arabic version of the Edinburgh Postnatal Depression Scale [EPDS] was used.** |
|  |  | (b) Indicate number of participants with missing data for each variable of interest |  | **N/A** |
|  |  | (c) *Cohort study*—Summarise follow-up time (eg, average and total amount) | 6 | **6 weeks** |
| Outcome data | 15* | *Cohort study*—Report numbers of outcome events or summary measures over time | 1,2 | **91 patients were enrolled. Epidural analgesia was administered to 48.4% of mothers during labour. Nearly two-thirds of mothers learned about EA via sources including family members and social media. However, more than half reported worries regarding epidural analgesia. Edinburgh Postnatal Depression Scale scores showed that 38 mothers (41.8%) likely had depressive symptoms within two days following delivery. Further, 35 (38.5%) met criteria for postpartum depression at six weeks postpartum. For both groups regardless use of analgesia, the mean Visual Analogue Scale score at two days postpartum was 4.16 ± 2.13. Data revealed no correlation between epidural analgesia use and Edinburgh Postnatal Depression Scale within two days and at six weeks postpartum. Multiple regression analysis showed Edinburgh Postnatal Depression Scale scores correlated with Visual Analogue Scale scores but not epidural analgesia use at 1-2 days postpartum.** |
|  |  | *Case-control study—*Report numbers in each exposure category, or summary measures of exposure |  |  |
|  |  | *Cross-sectional study—*Report numbers of outcome events or summary measures |  |  |
| Main results | 16 | (*a*) Give unadjusted estimates and, if applicable, confounder-adjusted estimates and their precision (eg, 95% confidence interval). Make clear which confounders were adjusted for and why they were included | 7 | **IBM SPSS Statistics version 26. Independent sample T-test and correlation test were used to measure the association between two variables with 95% CI. In addition, One-way ANNOVA test were used to measure the association between three variables with 95% CI. All tests had a cutoff of p-value equal or less than 0.05 for to be significant.** |
|  |  | (*b*) Report category boundaries when continuous variables were categorized |  |  |
|  |  | (*c*) If relevant, consider translating estimates of relative risk into absolute risk for a meaningful time period |  |  |

Continued on next page

| Other analyses | 17 | Report other analyses done—eg analyses of subgroups and interactions, and sensitivity analyses |  |  |
| --- | --- | --- | --- | --- |
| Discussion | | | | |
| Key results | 18 | Summarise key results with reference to study objectives | 2 | **In the final analysis, 91 patients were enrolled. Epidural analgesia was administered to 48.4% of mothers during labour. Nearly two-thirds of mothers learned about EA via sources including family members and social media. However, more than half reported worries regarding epidural analgesia. Edinburgh Postnatal Depression Scale scores showed that 38 mothers (41.8%) likely had depressive symptoms within two days following delivery. Further, 35 (38.5%) met criteria for postpartum depression at six weeks postpartum. For both groups regardless use of analgesia, the mean Visual Analogue Scale score at two days postpartum was 4.16 ± 2.13. Data revealed no correlation between epidural analgesia use and Edinburgh Postnatal Depression Scale within two days and at six weeks postpartum. Multiple regression analysis showed Edinburgh Postnatal Depression Scale scores correlated with Visual Analogue Scale scores but not epidural analgesia use at 1-2 days postpartum.** |
| Limitations | 19 | Discuss limitations of the study, taking into account sources of potential bias or imprecision. Discuss both direction and magnitude of any potential bias | 17 | **Nearly half of the participants were not included in the final analysis due to loss to follow-up, which resulted in a small sample size. Pain analysis is a subjective measure of pain. Lastly, the study only included patients of King Abdulaziz University Hospital, Jeddah; therefore, results cannot be generalised to the general population.** |
| Interpretation | 20 | Give a cautious overall interpretation of results considering objectives, limitations, multiplicity of analyses, results from similar studies, and other relevant evidence | 13,14 | **Give a cautious overall interpretation of results considering objectives, limitations, multiplicity of analyses, results from similar studies, and other relevant evidence**  **The current study aimed to assess the association between EA use and the incidence of PPD.**  **In total, 91 women were enrolled in the final analysis. Similar to studies conducted in Istanbul, the mean age of patients in this study was 30.38 ± 5.09 years [20, 25]. Two thirds of mothers were between 25 and 35 years. This was similar to a previously published Canadian study showed that more than two-thirds of participants were aged 18-35 years [26].**  **Importantly, 41.8% and 38.5% of EPDS scores reported in the current study were ≥ 10 at 1-2 days and 6 weeks postpartum, respectively. Kaur et al. concluded that percentages of patients with depressive symptoms at 3-days postpartum differed by 2% between mothers received combined spinal and epidural and mothers not received. At 6-weeks postpartum mothers who used analgesia more frequently reported depressive symptoms versus those who did not [27.7% and 16.9%, respectively] [27]. Moreover, the present study showed that at 6-weeks postpartum, EPDS scores demonstrated that 38.5% of mothers likely had PPD. Similarly, in a Chinese study, 48.6% of participants experienced PPD with a higher incidence among mothers not receiving EA [34.6%] [25]. Studies conducted in Canada and the USA reported incidences of PPD of 12.9% and 20%, respectively [26, 28]. Some variability in PPD incidence was observed, even among studies evaluating PPD using the same scale. Notably, PPD occurred despite the use of analgesia, indicating PPD is at least partially associated with other factors.** |
| Generalisability | 21 | Discuss the generalisability (external validity) of the study results |  |  |
| Other information | |  | | |
| Funding | 22 | Give the source of funding and the role of the funders for the present study and, if applicable, for the original study on which the present article is based |  | **N/A** |

*Give information separately for cases and controls in case-control studies and, if applicable, for exposed and unexposed groups in cohort and cross-sectional studies.

**Note:** An Explanation and Elaboration article discusses each checklist item and gives methodological background and published examples of transparent reporting. The STROBE checklist is best used in conjunction with this article (freely available on the Web sites of PLoS Medicine at http://www.plosmedicine.org/, Annals of Internal Medicine at http://www.annals.org/, and Epidemiology at http://www.epidem.com/). Information on the STROBE Initiative is available at www.strobe-statement.org.
